# Mental healthcare utilisation among individuals with colorectal cancer: population-based cohort studies

**DOI:** 10.1136/bmjonc-2024-000690

**Published:** 2025-04-01

**Authors:** Vicki Cheng, Eric C Sayre, Vienna Cheng, Jonathan M Loree, Sharlene Gill, Rachel A Murphy, Alyssa Howren, Mary A De Vera

**Affiliations:** 1 The Faculty of Pharmaceutical Sciences, The University of British Columbia, Vancouver, British Columbia, Canada; 2 Collaboration for Outcomes Research and Evaluation, Vancouver, British Columbia, Canada; 3 British Columbia Centre on Substance Use, Vancouver, British Columbia, Canada; 4 BC Cancer, Vancouver, British Columbia, Canada; 5 Division of Medical Oncology, The University of British Columbia Faculty of Medicine, Vancouver, British Columbia, Canada; 6 School of Population and Public Health, The University of British Columbia, Vancouver, British Columbia, Canada; 7 Department of Epidemiology and Population Health, Stanford University School of Medicine, Palo Alto, California, USA; 8 Centre for Health Evaluation and Outcome Sciences, Vancouver, British Columbia, Canada

**Keywords:** Colorectal cancer, Epidemiology, Psycho-oncology, Health Services Research

## Abstract

**Objective:**

Individuals with colorectal cancer (CRC) have an increased risk of mental disorders, yet mental healthcare utilisation has not been adequately examined. We evaluated mental healthcare utilisation and receipt of minimally adequate treatments for anxiety and/or depression among individuals with and without CRC.

**Methods and analysis:**

We used administrative health databases from British Columbia, Canada, comprised of individuals with CRC and individuals without CRC, matched (1:1 ratio) on age, sex and incident mental disorder(s) (ie, occurring after CRC diagnosis/matched date). Primary outcomes were minimally adequate antidepressant pharmacotherapy (≥84 days’ supply) and psychological (≥4 services) treatment.

**Results:**

Among individuals with CRC, 1462 had incident anxiety (mean age 64.6±12.5 years, 59.2% females), 4640 had incident depression (mean age 66.3±12.3 years, 51.2% females). Approximately one in four individuals with CRC were diagnosed with anxiety (23.4%) and/or depression (23.2%) in the first year after CRC diagnosis. Minimally adequate antidepressant pharmacotherapy (36.2%) and psychological treatment (15.9%) for anxiety were significantly lower in CRC patients than in those without CRC (pharmacotherapy adjusted OR (aOR) 0.74; 95% CI 0.61, 0.88; psychological treatment aOR 0.74; 95% CI 0.58, 0.95). Similar findings were observed for depression (pharmacotherapy aOR 0.81; 95% CI 0.74, 0.90). Among individuals with CRC, mental healthcare utilisation persisted up to 10 years post-mental disorder diagnosis.

**Conclusions:**

Individuals with CRC receive less mental health treatment for anxiety and/or depression, compared with those without CRC. Findings raise awareness for the need for ongoing mental healthcare throughout and beyond CRC.

**WHAT IS ALREADY KNOWN ON THIS TOPIC**

- The current literature suggests that individuals with colorectal cancer (CRC) are at an increased risk of anxiety and depression; however, their mental healthcare utilisation following these diagnoses has not been adequately studied.

**WHAT THIS STUDY ADDS**

- This study found that individuals with CRC diagnosed with anxiety and/or depression had significantly lower odds of receiving minimally adequate antidepressant pharmacotherapy and psychological treatment compared with those without CRC who were diagnosed with anxiety and/or depression. Mental healthcare utilisation among individuals with CRC persisted up to 10 years post-mental disorder diagnosis, reflecting the need for continuous mental healthcare in this population throughout and beyond CRC.

**HOW THIS STUDY MIGHT AFFECT RESEARCH, PRACTICE OR POLICY**

- Findings highlight disparities in mental health treatment engagement among patients with CRC and underscore the need for targeted interventions and integrated mental healthcare. Policy-makers and healthcare professionals should prioritise long-term mental healthcare as part of comprehensive CRC care.

## Introduction

 Globally, colorectal cancer (CRC) is the third most common malignancy and the second most common cause of cancer-related death in the world.^1^ The high incidence and mortality rates of CRC in both developed and developing countries underscore the global health burden posed by this malignancy.^2^ Canada had an estimated 25 200 new CRC cases and 9400 CRC-related deaths in 2024 alone.^3^

Corresponding to the increasing incidence of CRC worldwide, research has shown that compared with the general population, individuals with CRC are at a higher risk of mental disorders.^4^,^7^ ‘Mental disorders’, which include anxiety and depression, are defined by the American Psychiatric Association as a spectrum of mental conditions ranging from mild to severe, all characterised by clinically significant disturbances in cognition, emotional regulation or behaviour, which may manifest through various emotional and cognitive symptoms.^8^,^10^ A 2022 systematic review and meta-analysis evaluating the onset of anxiety and depression among individuals with CRC found a non-significant association between CRC and anxiety (pooled HR 1.67; 95% CI 0.88, 3.17) and a significant association between CRC and depression (pooled HR 1.78; 95% CI 1.23, 2.57).^11^

Despite the risk of anxiety and depression in CRC, previous research has not clearly assessed the receipt of treatment following diagnosis—specifically, pharmacological or psychological—of these mental disorders.^12 13^ Indeed, only a limited number of observational studies have investigated mental healthcare utilisation among individuals with cancer in general. A 2012 case–control study examining patterns of treatment for newly diagnosed anxiety and depression post-cancer diagnosis found that psychiatric medications were prescribed within the first 6 months following the initial cancer diagnosis in 1066 (14.6%) cases and 161 (1.1%) controls.^12^ A 2015 cohort study assessing the impact of a cancer diagnosis on the timing of antidepressant initiation found that individuals with cancer were 42% more likely to initiate antidepressant treatment than individuals without cancer (HR 1.42; 95% CI 1.20, 1.70), with the initiation period peaking between 12 weeks before and 16 weeks post-cancer diagnosis.^13^ Furthermore, although barriers to accessing mental healthcare among individuals with CRC, such as stigma and economic concerns, have been reported,^14^ it remains unclear whether there is a corresponding gap in mental healthcare utilisation. We aimed to conduct a focused evaluation of mental healthcare utilisation, specifically assessing pharmacological and psychological treatments among individuals with CRC with new onset of anxiety and/or depression after their cancer diagnosis, compared with individuals without CRC. This evaluation will allow us to identify potential disparities in mental healthcare utilisation in the context of a CRC diagnosis.

## Methods

### Study design, data source and source population

**Figure 1:**
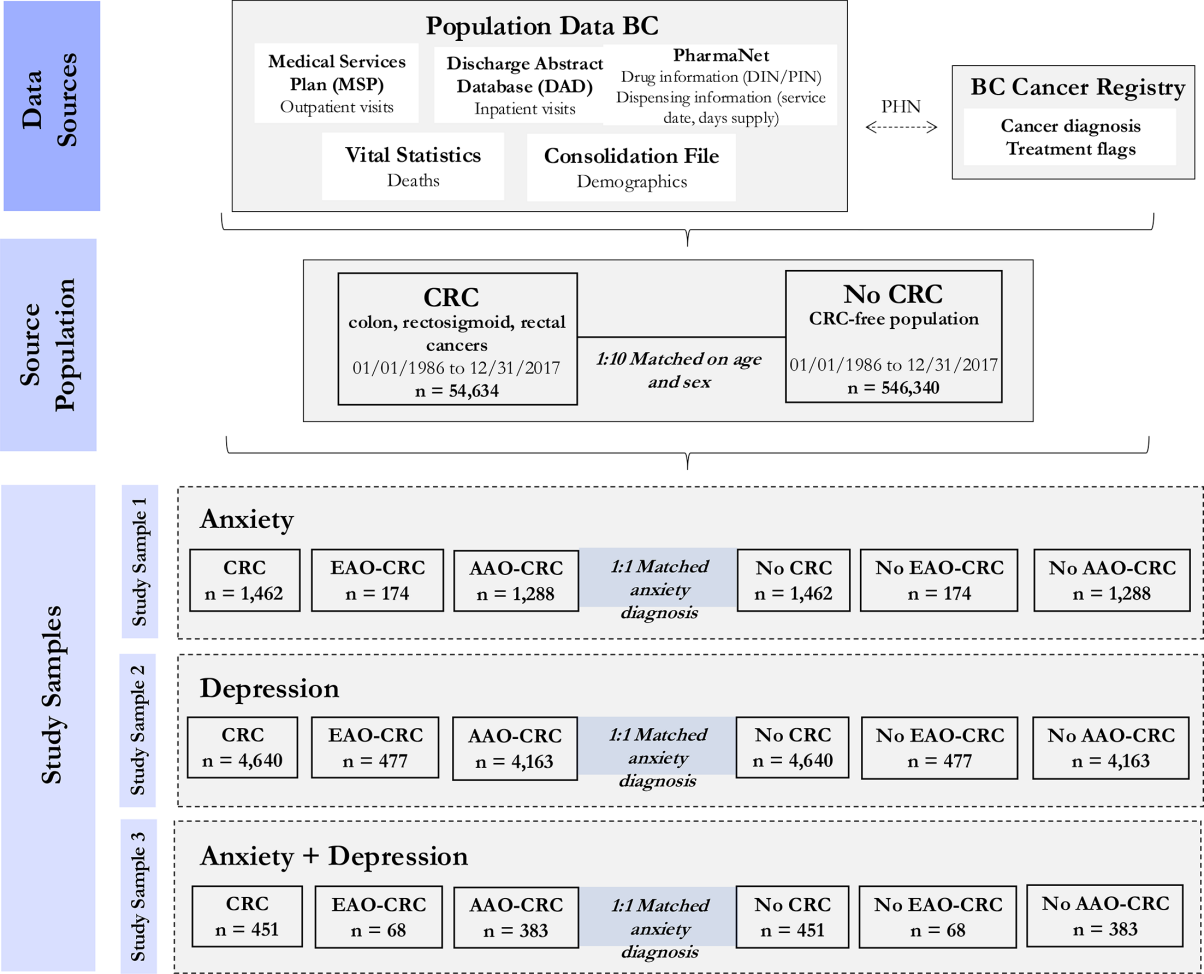
Overview of data sources, source population and study samples for individuals with colorectal cancer (CRC) and without CRC with anxiety, depression and anxiety+depression (dashed arrow shows linkages between databases using personal health numbers which are then deidentified). Due to the availability of PharmaNet data, the study samples consisted of individuals with CRC who were diagnosed with anxiety, depression and anxiety+depression from 1 January 1996 to 31 December 2018. AAO, average-age onset; DIN/PIN, drug identification number/product identification number; EAO, early-age onset; PHN, personal health number*.*

We conducted retrospective cohort studies using administrative health data from Population Data British Columbia (BC) which captures longitudinal, deidentified individual-level data for the province of British Columbia, Canada (5.6 million residents in 2024). This study design allowed us to examine pre-existing data over a defined time period to assess mental healthcare utilisation. The data holdings that were accessed for this study include: Medical Services Plan (outpatient visits), Discharge Abstract Database (inpatient visits), Vital Statistics File (deaths), Consolidation File (demographics) and PharmaNet (drug and dispensing information) since 1996. Using personal health numbers, we linked Population Data BC data to the BC Cancer Registry,^15^ which captures cancer diagnosis (date of diagnosis, tumour type and site) and treatment (treatment type and dates) information since 1986 (figure 1).

From these data sources, a source population was drawn which consisted of individuals diagnosed with CRC from 1 January 1986 to 31 December 2017 who were identified in the BC Cancer Registry using the International Classification of Diseases for Oncology (ICD-O), Third Edition codes: C18.3, C18.0, C18.2 (right colon); C18.6, C18.7, C18.5, C19 (left colon); C18.4 (transverse colon); C20, C21.8 (rectum); and C18.9, C18.1, C18.8 (unspecified). The aforementioned window of 1 January 1986 to 31 December 2017 allowed at least 5 years to have passed between a CRC diagnosis and analysis, ensuring that most patients received the majority of their treatment prior to the COVID-19 pandemic, which may have impacted access to care and experiences of anxiety and depression. Each individual with CRC was matched to individuals without cancer (1:10) on sex and age, with the date of CRC diagnosis assigned as the ‘matched date’. We further classified individuals as early-age onset CRC (EAO-CRC), for individuals who received their diagnosis at less than 50 years of age, and average-age onset CRC (AAO-CRC), for individuals who received their diagnosis at 50 years or greater. These data sources and source population have been used in prior population-based studies of CRC.^16^,^18^

### Study samples

**Figure 2:**
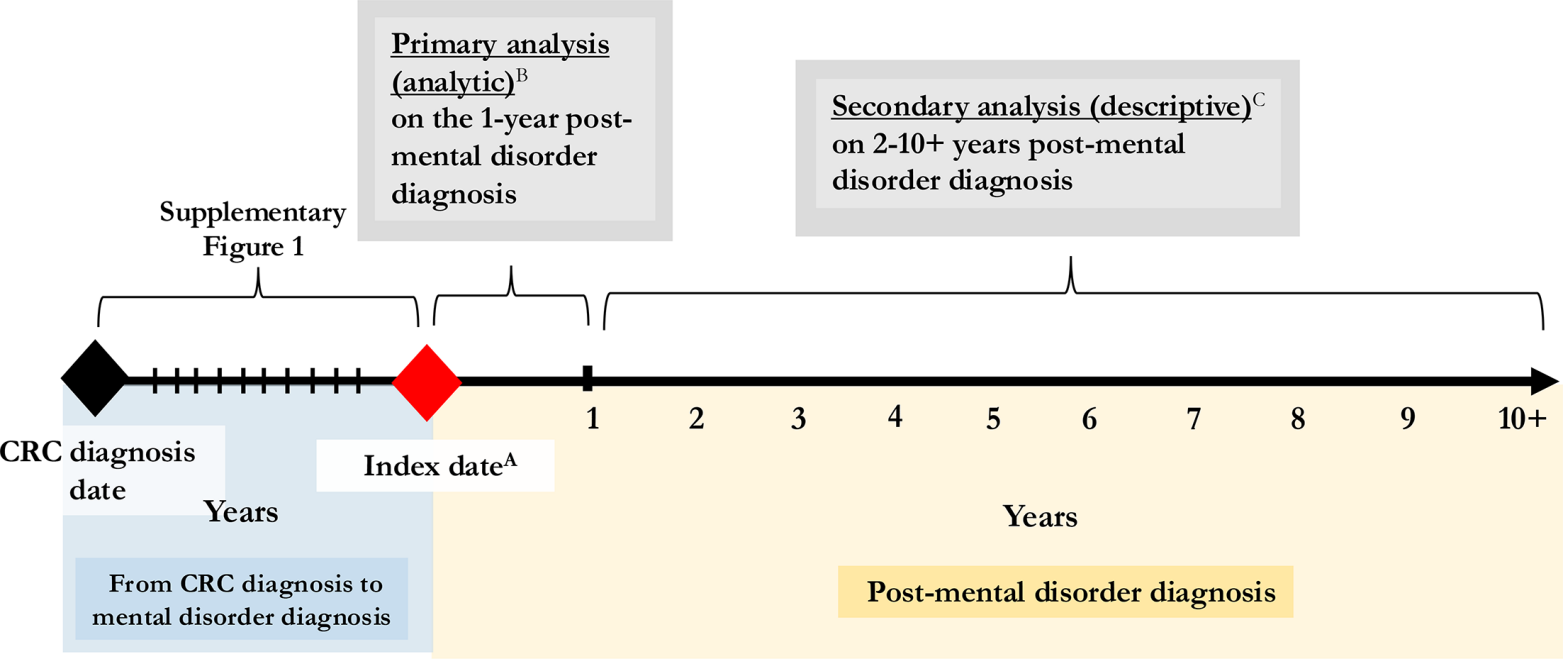
Study scheme. ^A^Index date=the date of a mental disorder diagnosis (anxiety, depression, anxiety+depression). ^B^Comparison of mental healthcare utilisation among individuals with and without CRC. ^C^Mid-term to long-term mental healthcare utilisation among individuals with CRC. CRC, colorectal cancer.

We created study samples, composed of individuals with CRC and individuals without CRC, matched (1:1 ratio) on age, sex and incident mental health disorder(s) (ie, occurring after CRC diagnosis/matched date) between 1 January 1996 and 31 December 2018. Start and end dates of mental disorder ascertainment correspond to the latest availability of relevant dataset (ie, PharmaNet) and end of follow-up. Mental disorders of interest were anxiety, depression and comorbid anxiety+depression (ie, individuals diagnosed with both anxiety and depression), which we defined using previously validated case definitions as follows. For anxiety, we required ≥1 inpatient visit (with ICD 10th Revision codes, F40–F41) or ≥2 outpatient visits (with ICD-9th Revision codes, 300.0, 300.2) within a 2-year period.^19 20^ For depression we required ≥1 inpatient visit (with ICD-10 codes, F20.4, F31.3–F31.5, F32.x, F33.x, F34.1, F41.2 and F43.2) or ≥2 outpatient visits (with ICD-9 codes, 296.2, 296.3, 296.5, 300.4, 309.x and 311) within a 1-year period.^21 22^ With our interest in mental healthcare utilisation in the context of CRC, we were interested in new onset mental disorder(s) after CRC diagnosis, which we refer to as incident anxiety, depression or anxiety+depression. Accordingly, we required that the date of the mental disorder diagnosis (defined as the index date) occur after the CRC diagnosis (matched date for CRC-free individuals). Individuals with CRC and without CRC who met the case definitions for anxiety and/or depression prior to the date of CRC diagnosis/matched date were excluded. Altogether, we had three study samples: (1) index anxiety (individuals with and without CRC matched on incident anxiety); (2) index depression (individuals with and without CRC matched on incident depression) and (3) index anxiety+depression (individuals with and without CRC matched on incident anxiety+depression) (figure 1). This study scheme is illustrated in figure 2, highlighting the temporal sequence, where the date of a mental disorder diagnosis (ie, index date) was required to occur after the CRC diagnosis/matched date.

### Outcome ascertainment

#### Pharmacotherapy

To identify pharmacotherapy, we used Anatomical Therapeutic Chemical (ATC) classification codes and Drug Identification Numbers/Product Identification Numbers (PIN) in PharmaNet—specifically for anxiolytics (ATC, N05B) and antidepressants (ATC, N06A). We evaluated classes overall as well as specific drugs (online supplemental table 1).online supplemental table 1

#### Psychological treatment

To identify publicly funded outpatient psychological treatments, including psychiatrist visits, counselling, psychotherapy, general practitioner mental health planning and telehealth service, we used fee-item codes from the BC Medical Services Commission Payment Schedule in the Medical Services Plan (online supplemental table 2).^23^

#### Hospitalisation

Inpatient visits were obtained from the Discharge Abstract Database, based on ICD-9/10 codes occurring in any diagnostic position, to determine the following outcomes: (1) hospitalisation for anxiety; (2) hospitalisation for depression and (3) hospitalisation for anxiety+depression.

#### Minimally adequate antidepressant pharmacotherapy and/or psychological treatment

Minimally adequate antidepressant pharmacotherapy for anxiety and depression was defined as antidepressant prescriptions filled with a supply of ≥84 days,^9 24 25^ within 1 year following the index date for anxiety and/or depression, based on prior literature that assessed minimally adequate care for depression.^18 24^ For depression, this corresponds to the 12-week acute treatment phase, during which individuals initiating antidepressant therapy are expected to be on medication.^25^ For anxiety, the first-line pharmacotherapies primarily consist of antidepressants for most cases of anxiety disorders, except for specific phobias, where pharmacotherapy is not indicated.^9^ Finally, given that anxiolytics are generally prescribed for short-term use, they were excluded from our definition of minimally adequate pharmacotherapy in this study.^9^

Minimally adequate psychological treatment was defined as receiving ≥4 psychiatrist, counselling, psychotherapy or telehealth sessions within 1 year following the index date for anxiety and/or depression, based on prior literature and the acceptable minimum coverage within the publicly funded healthcare system in British Columbia.^18 24^

### Covariate assessment

We considered as covariates: age, sex (ie, biological construct, which is recorded in the Consolidation file), neighbourhood income quintile (Quintile of Adjusted Income Per Person Equivalent; 1–5: a measure of area-based income adjusted for household size), residence (urban vs rural) and comorbidities, represented by the Charlson–Romano Comorbidity Index.^26^

### Statistical analysis

We used descriptive statistics to evaluate the sociodemographic characteristics of individuals with CRC and without CRC across the three study samples (ie, index anxiety, index depression, index anxiety+depression).

In our primary evaluation of mental healthcare utilisation, we compared individuals with CRC and without CRC, focusing on the 1-year period following the index date as this is the critical period for treatment after a mental disorder diagnosis (figure 2).^24 25^ We computed the proportion of individuals with ≥1 encounter (eg, filling a prescription for pharmacotherapy, having a psychological treatment) and compared individuals with and without CRC using the Pearson χ^2^ test/Fisher’s exact test. We also computed counts of the encounters and compared individuals with and without CRC using the Wilcoxon rank-sum test. For antidepressants, we also evaluated the mean days of supply and the proportion of days covered (ie, calculated as the number of days covered from the initial prescription date to the end of the 1-year period). Finally, we conducted a sensitivity analysis to exclude anxiolytics that may have been prescribed for as-needed use (eg, to address side effects of CRC treatment rather than to treat a mental disorder).^27^ We defined this as fewer than 26 anxiolytics dispensed over the 1-year period (ie, less than once every 2 weeks) based on our team’s clinical expertise.^9 25 27^ All analyses were stratified by age at CRC diagnosis (ie, EAO-CRC and AAO-CRC). Multivariable logistic regression models, adjusted for age, sex, comorbidities, income and residence, were used to evaluate the odds of individuals with CRC receiving minimally adequate antidepressant pharmacological or psychological care for their index anxiety and/or depression as compared with individuals without CRC. All multivariable models were also stratified by age at CRC diagnosis as well as by sex.

In our secondary descriptive analyses, we assessed mental healthcare utilisation among individuals with CRC beyond the 1-year period following the index date, up to 10 years (figure 2). Such analyses allowed us to examine the mid-term to long-term mental healthcare utilisation among individuals with CRC. The specific outcomes in these analyses included pharmacotherapy (anxiolytics, antidepressants) and psychological treatments (publicly funded counselling and psychotherapy). All analyses were conducted using SAS statistical software V.9.4 (SAS Institute).

### Study conduct

Access to data provided by the Data Stewards is subject to approval but can be requested for research projects through the Data Stewards or their designated service providers. The following data sets were used in this study: Medical Services Plan, Discharge Abstract Database, PharmaNet, Consolidation File, Vital Statistics File and British Columbia Cancer Registry. You can find further information regarding these data sets by visiting the PopData project webpage at: https://my.popdata.bc.ca/project_listings/18-088/collection_approval_dates. All inferences, opinions and conclusions drawn in this publication are those of the author(s) and do not reflect the opinions or policies of the Data Steward(s).

### Patient and public involvement

Patients or the public were not involved in the design, or conduct, or reporting, or dissemination plans of our research.

## Results

### Study samples

Characteristics of study samples of individuals with CRC and no CRC matched on incident (1) anxiety, (2) depression and (3) anxiety+depression are summarised in table 1. Altogether, 1462 individuals with CRC had index anxiety, 4640 had index depression and 451 had index anxiety and depression. The median time from CRC diagnosis to a mental disorder diagnosis, for index anxiety and index depression, was 3.8 and 3.9 years, respectively. Individuals with CRC were largely diagnosed with anxiety (23.4%) and/or depression (23.2%) in the first year after CRC diagnosis, though we also observed diagnosis of mental disorders in subsequent years (online supplemental figure 1). Online supplemental tables 3 and 4 show characteristics of study samples according to age of CRC diagnosis (ie, EAO-CRC and AAO-CRC).

**Table 1:** Characteristics of study samples of individuals with colorectal cancer (CRC) and no CRC matched on incident 1 anxiety, 2 depression and 3 anxiety+depression

	Study sample 1Anxiety		Study sample 2Depression		Study sample 3Anxiety+depression	
**Characteristic**	**CRC**(**n=1462**)	**No CRC**(**n=1462**)	**CRC**(**n=4640**)	**No CRC**(**n=4640**)	**CRC**(**n=451**)	**No CRC (n=451**)
Years from CRC diagnosis to mental disorder diagnosis, median	3.8	3.9	3.7	3.7	7.2	7.2
Demographic factors						
Age, mean (SD)	64.6 (12.5)	64.6 (12.5)	66.3 (12.3)	66.3 (12.3)	64.0 (13.2)	64.0 (13.2)
Sex, n (%)						
Male	596 (40.8)	596 (40.8)	2264 (48.8)	2264 (48.8)	178 (39.5)	178 (39.5)
Female	866 (59.2)	866 (59.2)	2376 (51.2)	2376 (51.2)	273 (69.5)	273 (69.5)
Neighbourhood income quintile, n (%)						
Quintile 1	335 (22.9)	332 (22.7)	1026 (22.1)	1075 (23.2)	110 (24.4)	94 (20.8)
Quintile 2	377 (19.0)	260 (17.8)	887 (19.1)	888 (19.1)	85 (18.9)	98 (21.7)
Quintile 3	299 (20.4)	297 (20.3)	937 (20.2)	896 (19.3)	77 (17.1)	82 (18.2)
Quintile 4	277 (19.0)	289 (20.0)	884 (19.1)	929 (20.0)	86 (19.1)	90 (20.0)
Quintile 5	274 (18.7)	284 (19.4)	906 (19.5)	852 (18.4)	93 (20.6)	87 (19.3)
Residence, n (%)						
Urban	1264 (86.5)	1264 (86.5)	4034 (86.9)	4006 (86.3)	398 (88.3)	384 (85.1)
Rural	198 (13.5)	198 (13.5)	606 (13.1)	634 (13.7)	53 (11.8)	67 (14.9)
Healthcare utilisation						
Outpatient visits, mean (SD)	15.9 (12.1)	13.1 (13.1)	14.6 (10.8)	11.6 (11.9)	15.7 (10.0)	14.6 (14.6)
Comorbidities						
Charlson-Romano Comorbidity Index, mean (SD)*	1.8 (2.5)	0.3 (0.9)	1.8 (2.4)	0.3 (0.8)	1.7 (2.5)	0.3 (0.7)
CRC characteristics						
Site, n (%)						
Left colon	612 (41.9)	---	1947 (42.0)	---	182 (40.4)	---
Right colon	224 (15.3)	---	619 (13.3)	---	70 (15.5)	---
Transverse colon	83 (5.7)	---	272 (5.9)	---	32 (7.1)	---
Unspecified	51 (3.5)	---	183 (3.9)	---	19 (4.2)	---
Rectum	492 (33.7)	---	1619 (34.9)	---	148 (32.8)	---
Treatment, n (%)						
Surgery	761 (52.1)	---	2242 (48.3)	---	229 (50.8)	---
Chemotherapy	628 (43.0)	---	1823 (39.3)	---	174 (38.6)	---
Radiation	331 (22.6)	---	1086 (23.4)	---	94 (20.8)	---
Stage†, n (%)	(n=312)		(n=583)		(n=58)	
1	67 (21.5)	---	112 (19.2)	---	12 (20.7)	---
2	79 (23.5)	---	165 (28.3)	---	13 (22.4)	---
3	120 (38.5)	---	217 (37.2)	---	25 (43.1)	---
4	46 (14.7)	---	89 (15.3)	---	8 (13.8)	---

Descriptive statistics were determined for the year prior to CRC diagnosis date/matched date.

* All comorbidities except for cancer.

† Stage data are available from 2010 onwards.

CRCcolorectal cancer

### Primary analysis: comparison of mental healthcare utilisation among individuals with and without CRC

Mental healthcare utilisation—pharmacotherapy, psychological treatment and hospitalisation—in the first year after index mental disorder diagnosis are summarised in table 2 (eg, reported as proportion of individuals with and without CRC) and online supplemental table 5 (eg, reported as number (mean, SD) of encounters). Receipt of minimally adequate mental healthcare in terms of antidepressant pharmacotherapy and psychological treatment are summarised in online supplemental table 6. Finally, table 3 summarises multivariable logistic regression models evaluating the receipt of minimally adequate pharmacotherapy and psychological treatment in study samples of individuals with CRC and no CRC. Online supplemental tables 7 - 12 show the mental healthcare utilisation (including proportion of individuals with and without CRC and number (mean, SD) of encounters) of study samples stratified according to age of CRC diagnosis (ie, EAO-CRC and AAO-CRC).

**Table 2:** Utilisation of mental healthcare in study samples of individuals with CRC and no CRC with 1 anxiety, 2 depression and 3 anxiety+depression reported as proportion among individuals

Outcome	Study sample 1Anxiety			Study sample 2Depression			Study sample 3Anxiety+depression		
	CRC	No CRC	P value	CRC	No CRC	P value	CRC	No CRC	P value
Pharmacotherapy, n (%)									
Anxiolytics									
All anxiolytics	493 (45.7)	484 (40.1)	**<0.05**	1105 (33.0)	1159 (30.7)	**<0.05**	161 (48.8)	177 (47.5)	0.76
By class									
Benzodiazepines	481 (44.6)	479 (39.7)	**<0.05**	1055 (31.5)	1102 (29.2)	**<0.05**	158 (47.9)	169 (45.3)	0.50
Hydroxyzine	17 (1.6)	24 (2.0)	0.53	78 (2.3)	79 (2.1)	0.52	11 (3.3)	7 (1.9)	0.63
Buspirone	6 (0.6)	6 (0.5)	1.00	5 (0.1)	17 (0.5)	**<0.05**	<5	5 (1.3)	0.22
Antidepressants									
All antidepressants	486 (45.1)	625 (51.8)	**<0.05**	1974 (58.9)	2360 (62.5)	**<0.05**	219 (66.4)	268 (71.8)	0.12
By class									
Selective serotonin reuptake inhibitors	334 (31.0)	397 (32.9)	0.35	1375 (41.0)	1714 (45.4)	**<0.05**	161 (48.8)	157 (42.1)	0.08
Non-selective monoamine reuptake inhibitors†	86 (8.0)	102 (8.5)	0.70	278 (8.3)	327 (8.7)	0.61	33 (10.0)	39 (10.5)	0.90
Other‡	198 (18.4)	281 (23.3)	**<0.05**	743 (22.2)	865 (22.9)	0.48	88 (26.7)	153 (41.0)	**<0.05**
Psychological treatment, n (%)									
By service type									
Psychiatrist	93 (8.6)	148 (12.3)	**<0.05**	455 (13.6)	508 (13.4)	0.89	48 (14.5)	66 (17.7)	0.26
Publicly funded counselling	474 (44.0)	490 (40.6)	0.11	1696 (50.6)	1743 (46.1)	**<0.05**	156 (47.3)	151 (40.5)	0.08
Publicly funded psychotherapy	142 (13.2)	209 (17.3)	**<0.05**	601 (17.9)	629 (16.7)	0.16	77 (23.3)	104 (27.9)	0.19
Telehealth	<5*	<5*	1.00	<5*	<5*	1.00	<5*	0	0.22
Mental health planning	9 (0.8)	9 (0.7)	0.82	35 (1.0)	40 (1.1)	1.00	6 (1.8)	5 (1.3)	0.76
Hospitalisations, n (%)									
For anxiety	289 (26.8)	263 (21.8)	**<0.05**	55 (1.6)	61 (1.6)	0.93	97 (29.4)	86 (23.1)	0.06
For depression	93 (8.6)	118 (9.8)	0.35	474 (14.1)	447 (11.8)	**<0.05**	69 (20.9)	69 (18.5)	0.45
For anxiety+depression	74 (6.9)	103 (8.5)	0.16	37 (1.1)	55 (1.5)	0.21	45 (13.6)	49 (13.1)	0.91

Bolded values indicate statistical significance at p<0.05.

* Cell sizes <5 are not reported according to agreements of the data access request.

† Include tricyclic antidepressants.

‡ Other antidepressants included selective serotonin-norepinephrine reuptake inhibitors, trazodone and mirtazapine.

CRCcolorectal cancer

**Table 3:** Multivariable logistic regression models evaluating the receipt of minimally adequate pharmacotherapy and psychological treatment in study samples of individuals with CRC and no CRC with 1 anxiety, 2 depression and 3 anxiety+depression

	Study sample 1Anxiety		Study sample 2Depression		Study sample 3Anxiety+	
	**OR (95% CI**)		**OR (95% CI**)		**OR (95% CI**)	
Minimally adequate antidepressant pharmacotherapy						
Unadjusted model	0.78 (0.66, 0.93)		0.83 (0.76, 0.91)		0.81 (0.60, 1.09)	
Adjusted model*	0.74 (0.61, 0.88)		0.81 (0.74, 0.90)		0.84 (0.60, 1.16)	
	Males	Females	Males	Females	Males	Females
Unadjusted model	0.94 (0.72, 1.24)	0.69 (0.56, 0.86)	0.82 (0.71, 0.93)	0.84 (0.74, 0.96)	1.02 (0.62, 1.69)	0.70 (0.48, 1.03)
Adjusted model†	0.89 (0.65, 1.19)	0.66 (0.52, 0.84)	0.81 (0.70, 0.94)	0.82 (0.72, 0.95)	1.16 (0.67, 2.02)	0.70 (0.47, 1.05)
	EAO-CRC	AAO-CRC	EAO-CRC	AAO-CRC	EAO-CRC	AAO-CRC
Unadjusted model	1.13 (0.70, 1.80)	0.74 (0.62, 0.89)	0.64 (0.49, 0.85)	0.86 (0.78, 0.95)	0.54 (0.25, 1.15)	0.87 (0.63, 1.21)
Adjusted model‡	1.21 (0.71, 2.07)	0.69 (0.56, 0.83)	0.60 (0.43, 0.82)	0.85 (0.76, 0.94)	0.57 (0.24, 1.36)	0.89 (0.63, 1.27)
Minimally adequate psychological treatment						
Unadjusted model	0.88 (0.71, 1.10)		1.10 (0.98, 1.24)		0.88 (0.62, 1.24)	
Adjusted model*	0.74 (0.58, 0.95)		0.97 (0.85, 1.11)		0.74 (0.51, 1.08)	
	Males	Females	Males	Females	Males	Females
Unadjusted model	0.92 (0.64, 1.31)	0.86 (0.65, 1.14)	1.04 (0.88, 1.22)	1.18 (1.00, 1.39)	1.09 (0.61, 1.93)	0.78 (0.51, 1.20)
Adjusted model†	0.82 (0.55, 1.21)	0.69 (0.50, 0.95)	0.92 (0.77, 1.11)	1.02 (0.85, 1.23)	0.97 (0.52, 1.82)	0.65 (0.40, 1.03)
	EAO-CRC	AAO-CRC	EAO-CRC	AAO-CRC	EAO-CRC	AAO-CRC
Unadjusted model	0.75 (0.42, 1.33)	0.91 (0.71, 1.16)	0.98 (0.71, 1.34)	1.12 (0.99, 1.27)	0.65 (0.28, 1.49)	0.94 (0.65, 1.37)
Adjusted model‡	0.60 (0.30, 1.20)	0.76 (0.58, 0.99)	0.86 (0.59, 1.25)	0.99 (0.86, 1.14)	0.55 (0.20, 1.48)	0.80 (0.53, 1.21)
Minimally adequate antidepressant pharmacotherapy OR psychological treatment						
Unadjusted model	0.77 (0.65, 0.90)		0.89 (0.81, 0.98)		0.72 (0.53, 0.99)	
Adjusted model*	0.69 (0.57, 0.83)		0.83 (0.75, 0.92)		0.70 (0.50, 0.98)	
	Males	Females	Males	Females	Males	Females
Unadjusted model	0.89 (9.68, 1.16)	0.69 (0.56, 0.86)	0.85 (0.74, 0.97)	0.93 (0.81, 1.05)	1.09 (0.65, 1.83)	0.56 (0.37, 0.83)
Adjusted model†	0.77 (0.58, 1.04)	0.65 (0.51, 0.81)	0.79 (0.68, 0.91)	0.88 (0.76, 1.01)	1.09 (0.62, 1.92)	0.54 (0.35, 0.83)
	EAO-CRC	AAO-CRC	EAO-CRC	AAO-CRC	EAO-CRC	AAO-CRC
Unadjusted model	0.94 (0.59, 1.49)	0.74 (0.62, 0.89)	0.72 (0.54, 0.95)	0.91 (0.83, 1.01)	0.42 (0.18, 0.94)	0.80 (0.57, 1.12)
Adjusted model‡	1.00 (0.59, 1.71)	0.65 (0.54, 0.79)	0.65 (0.47, 0.90)	0.86 (0.77, 0.96)	0.39 (0.15, 1.02)	0.76 (0.53, 1.10)
Minimally adequate antidepressant pharmacotherapy and psychological treatment						
Unadjusted model	0.91 (0.70, 1.18)		0.98 (0.84, 1.14)		0.98 (0.67, 1.44)	
Adjusted model*	0.80 (0.60, 1.07)		0.89 (0.75, 1.05)		0.90 (0.59, 1.35)	
	Males	Females	Males	Females	Males	Females
Unadjusted model	1.04 (0.68, 1.60)	0.83 (0.60, 1.16)	0.96 (0.77, 1.19)	1.00 (0.81, 1.24)	1.01 (0.54, 1.90)	0.96 (0.60, 1.56)
Adjusted model†	1.07 (0.67, 1.71)	0.67 (0.46, 0.97)	0.93 (0.73, 1.18)	0.86 (0.68, 1.09)	1.06 (0.53, 2.11)	0.82 (0.49, 1.38)
	EAO-CRC	AAO-CRC	EAO-CRC	AAO-CRC	EAO-CRC	AAO-CRC
Unadjusted model	0.97 (0.49, 1.94)	0.90 (0.68, 1.19)	0.74 (0.48, 1.13)	1.02 (0.87, 1.20)	0.73 (0.29, 1.87)	1.04 (0.68, 1.59)
Adjusted model‡	0.75 (0.33, 1.70)	0.80 (0.59, 1.09)	0.61 (0.36, 1.02)	0.93 (0.78, 1.11)	0.68 (0.23, 2.03)	0.97 (0.62, 1.51)

* Adjusted for age, sex, neighbourhood income quintile, residence and Charlson-Romano Comorbidity Index.

† Adjusted for age, neighbourhood income quintile, residence and Charlson-Romano Comorbidity Index.

‡ Adjusted for sex, neighbourhood income quintile, residence and Charlson-Romano Comorbidity Index.

AAO-CRCaverage-age onset colorectal cancerEAO-CRCearly-age onset CRC

### Study sample 1: anxiety

#### Pharmacotherapy

A higher proportion of individuals with CRC (45.7%) had ≥1 anxiolytic prescription dispensed compared with those without CRC (no CRC: 40.1%, p<0.05). In contrast, 45.1% of individuals with CRC had ≥1 antidepressant prescription dispensed, which was lower than those without CRC (51.8%, p<0.05) (table 2).

#### Psychological treatment

Fewer individuals with CRC (8.6%) received ≥1 psychiatrist service, compared with those without CRC (12.3%, p<0.05). Similarly, 13.2% of individuals with CRC received ≥1 publicly funded psychotherapy, which was lower than individuals without CRC (17.3%, p<0.05) (table 2).

#### Hospitalisation

The proportion of individuals with CRC with index anxiety having ≥1 hospitalisation for anxiety (26.8%) was higher than those with no CRC with index anxiety (21.8%, p<0.05) (table 2).

#### Minimally adequate antidepressant pharmacotherapy and/or psychological treatment

Approximately one-third of individuals with CRC with index anxiety (36.2%) received minimally adequate antidepressant pharmacotherapy, which was lower compared with those without CRC with index anxiety (42.0%, p<0.05) (online supplemental table 6). Multivariable logistic regression models indicate that individuals with CRC had lower odds of receiving minimally adequate antidepressant pharmacotherapy (adjusted OR (aOR) 0.74; 95% CI 0.61, 0.88) and minimally adequate psychological treatment (aOR 0.74; 95% CI 0.58, 0.95) compared with those without CRC. In sex-stratified analysis, among females, those with CRC had 34% lower odds of receiving minimally adequate antidepressant pharmacotherapy and 31% lower odds of receiving minimally adequate psychological treatment, compared with those without CRC. In age-stratified analysis, among individuals with AAO-CRC, those with AAO-CRC had 31% lower odds of receiving minimally adequate antidepressant pharmacotherapy and 24% lower odds of minimally adequate psychological treatment, compared with those without AAO-CRC (table 3).

### Study sample 2: depression pharmacotherapy

Approximately 33% of individuals with CRC with index depression had ≥1 anxiolytic prescription dispensed, which was higher compared with those without CRC with index depression (30.7%, p<0.05). In contrast, 58.9% of individuals with CRC had ≥1 antidepressant prescription dispensed, lower than those without CRC (62.5%, p<0.05) (table 2).

#### Psychological treatment

The proportion of individuals with depression having ≥1 publicly funded counselling was higher for those with CRC (50.6%) compared with those with no CRC (46.1%, p<0.05) (table 2).

#### Hospitalisation

The proportion of individuals with ≥1 hospitalisation for depression was higher for individuals with CRC (14.1%) compared with those with no CRC (11.8%, p<0.05) (table 2).

#### Minimally adequate antidepressant pharmacotherapy and/or psychological treatment

Less than half of individuals with CRC (46.3%) received minimally adequate antidepressant pharmacotherapy, lower than those without CRC (50.9%, p<0.05) (online supplemental table 6). Multivariable logistic regression models indicate that individuals with CRC had 19% lower odds of receiving minimally adequate antidepressant pharmacotherapy (aOR 0.81; 95% CI 0.74, 0.90). In sex-stratified analysis, among females, those with CRC had 18% lower odds of receiving minimally adequate antidepressant pharmacotherapy, compared with those without CRC. Among males, those with CRC had 19% lower odds of receiving minimally adequate antidepressant pharmacotherapy, compared with those without CRC. In the age-stratified analysis, among individuals with EAO-CRC, those with EAO-CRC had 40% lower odds of receiving minimally adequate antidepressant pharmacotherapy, compared with those without EAO-CRC. Among individuals with AAO-CRC, those with AAO-CRC had 15% lower odds of receiving minimally adequate antidepressant pharmacotherapy, compared with those without AAO-CRC (table 3).

A sensitivity analysis excluding individuals using anxiolytics for as-needed purposes (eg, 1–2 tablets before chemotherapy) showed a 5% reduction in the proportion of individuals with ≥1 anxiolytic prescription dispensed in both study samples 1 (anxiety) and 2 (depression), while findings remained significant. The sensitivity analysis suggests that anxiolytic prescriptions dispensed were primarily to treat diagnosed anxiety, rather than for as-needed use to manage potential cancer treatment side effects (eg, as an antiemetic).

### Secondary analysis: mid-term to long-term mental healthcare utilisation among individuals with CRC

Mid-term to long-term mental healthcare utilisation—pharmacotherapy, psychological treatment and hospitalisation—among individuals with CRC and stratified by EAO-CRC and AAO-CRC from years 2 to 10 after a mental disorder diagnosis are shown in onlinesupplemental figures 2^4^. Finally, online supplemental figure 5 presents a subgroup analysis by non-metastatic CRC (stages 1–3) at CRC diagnosis, focusing on the pharmacotherapy mental healthcare utilisation, specifically antidepressant use.

In the sample with index anxiety, 32% of individuals with CRC continued using anxiolytics and 40% used antidepressants 5 years after their anxiety diagnosis. At 10 years, 30% still used anxiolytics and 34% used antidepressants. With respect to psychological treatment, counselling was the most common, accessed by 20% of CRC individuals at 5 years and 15% at 10 years. In the sample with index depression, 21% of individuals with CRC continued using anxiolytics and 43% used antidepressants at 5 years. At 10 years, 10% of CRC individuals used anxiolytics and 38% used antidepressants. Counselling remained the most used psychological treatment, accessed by 18% of individuals with CRC at 5 years and 15% at 10 years postdepression diagnosis (online supplemental figure 2).

Lastly, a subgroup analysis of mental healthcare utilisation, specifically antidepressant use, among non-metastatic CRC (stages 1–3) at CRC diagnosis revealed that individuals diagnosed with stages 1–3 CRC had a high proportion and sustained antidepressant utilisation from 1 to 5 years after a mental disorder diagnosis (online supplemental figure 5). Data for stage four individuals were unavailable beyond 2 years after a mental disorder diagnosis; thus, they were not included in this subgroup analysis.

## Discussion

Our population-based study highlights substantial underutilisation of mental healthcare in individuals with CRC, with fewer than half receiving minimally adequate antidepressant treatment of ≥84 days and less than 20% receiving ≥4 psychological services in the year following mental disorder diagnosis. Compared with individuals without CRC, those with CRC had significantly lower odds of receiving minimally adequate pharmacological or psychological treatment for anxiety, with similar results for depression, though not statistically significant for minimally adequate psychological treatment. We also found notable differences in the use of anxiolytics, antidepressants (eg, selective serotonin reuptake inhibitors (SSRIs), benzodiazepines) and mental disorder-related hospitalisations. Mental healthcare utilisation persisted up to 10 years postmental disorder diagnosis in individuals with CRC, underscoring the need for continuous mental healthcare in this population throughout and beyond CRC.

Research has shown that following a diagnosis of cancer, individuals are at an increased risk of common mental disorders, such as anxiety and depression.^28^,^30^ A 2022 systematic review and meta-analysis found that specifically after a CRC diagnosis, individuals have a 51% increased risk of depression and a 43% increased risk of anxiety.^11^ Aside from representing important mental health concerns in cancer overall and specifically among those with CRC, the literature reveals that these disorders are strongly related and often co-occur,^31 32^ share overlapping symptoms,^33^ exhibit common genetic predispositions^34^ and commonly transition between each other over a lifetime.^35 36^ Despite their high comorbidity, anxiety and depression are recognised as distinct conditions due to evidence supporting their unique phenotypes.^37 38^ Our study reflects the complexity of these disorders with study samples representing those with anxiety, depression and comorbid anxiety+depression, to capture their distinct and combined impacts on mental healthcare utilisation.

Following a mental disorder diagnosis, timely and effective treatment for anxiety and/or depression is crucial, especially for individuals with CRC, as a mental disorder is linked to higher mortality (HR 2.18; 95% CI 2.02, 2.35),^5^ and as shown in our present study, greater hospitalisations due to either anxiety and/or depression. While some studies have examined mental healthcare in patients with cancer, few have focused specifically on CRC. A 2005 cross-sectional study by Kadan-Lottick *et al* found that only 45% of patients with advanced cancer with psychiatric disorders received mental health services, including anxiolytics and antidepressants,^39^ which aligns with our findings. A 2024 cohort study in the USA found significant differences in the proportion of pharmacotherapy use within 3 years after a mental disorder diagnosis between individuals with and without pancreatic cancer who had diagnosed anxiety or depression. Specifically, 52.7% of those with pancreatic cancer and diagnosed anxiety used pharmacotherapy, as did 39.7% of those with pancreatic cancer and diagnosed depression.^40^ Similarly, our findings show that less than half of individuals with CRC diagnosed with anxiety and/or depression used pharmacotherapy in the year after a mental disorder diagnosis.

Although previous studies have examined minimally adequate pharmacotherapy, definitions vary, including appropriate dose,^41^,^43^ appropriate duration^43^ or at least one dispensed prescription.^44^ Walker *et al* conducted a cross-sectional study of 21 151 patients with cancer, including CRC, and reported that 73% of patients with cancer diagnosed with depression were not receiving any effective treatment for depression, with less than 25% receiving an antidepressant drug at a minimal effective dose.^41^ Our use of Canadian clinical guidelines for anxiety and depression^9 25^ to define minimally adequate antidepressant pharmacotherapy (≥84 days’ supply) adds new insights into whether individuals with CRC are receiving appropriate mental healthcare for their incident mental disorder(s). Indeed, defining minimally adequate antidepressant pharmacotherapy as ≥84 days’ supply aligns with previous research^2445^,^47^; however, to our knowledge, no studies have specifically evaluated this in individuals with cancer after a mental disorder diagnosis. Thus, our study is among the first to quantify the extent of minimally adequate antidepressant pharmacotherapy in this population, offering a broader understanding of mental healthcare utilisation following a CRC diagnosis. Given that we observed individuals with CRC and anxiety had significantly lower odds of receiving minimally adequate antidepressant pharmacotherapy (aOR 0.74; 95% CI 0.61, 0.88) and psychological treatment (aOR 0.74; 95% CI 0.58, 0.95), as did those with depression, compared with individuals without CRC, our findings underscore the need for continuous access to and prioritisation of mental healthcare for individuals with CRC in Canada. This may include interventions to address potential barriers such as stigma,^48^ low perceived needs^49^ and limited healthcare provider awareness, and gaps in integration between oncology and mental health services.^50^

Our present study, conducted within the public healthcare system of BC, defined minimally adequate psychological treatment as receiving ≥4 psychological treatment visits.^18 24^ In the USA, studies on Medicaid-enrolled individuals defined minimally adequate psychotherapy as four visits within 12 weeks of a mental disorder treatment episode.^45^,^47^ While Canadian studies have also considered ≥4 psychological treatment visits as minimally adequate,^24 44 51^ to our knowledge, none have specifically examined this in patients with cancer. Our findings indicate that compared with individuals without CRC, only 15.9% of individuals with CRC received minimally adequate psychological treatment in the 1 year after an anxiety diagnosis, while 20.1% received this after a depression diagnosis. Although these proportions are low and non-significant, this undertreatment may be due to our study’s use of population-based data from BC, which does not capture privately funded mental health services. This lack of data is notable as approximately 30% of Canadians pay out of pocket for privately funded mental health services.^50^

While our findings indicate that the highest rate of mental healthcare utilisation occurred within the first year after a mental disorder diagnosis, they also reveal that individuals with CRC continue to use mental health treatments in the mid term to long term, highlighting the ongoing impact that mental health has on patients even into the mid-term to long-term (ie, ‘survivorship’^52^) phase of CRC care. We also observed that individuals with CRC continue to receive clinical diagnoses of anxiety and/or depression and use mental healthcare services in the 2–10 years after their mental disorder diagnoses, which is consistent with prior studies.^5 7 53^ A 2019 cohort study by Lloyd *et al* found that after a CRC diagnosis, individuals with CRC were at increased risk for any mental disorder diagnosis even at >5 years after a CRC diagnosis (HR 1.20; 95% CI 1.07, 1.36).^5^ Similarly, our findings show that approximately 18% to 43% of patients with CRC diagnosed with anxiety and/or depression continued using anxiolytics and antidepressants at 5 and 10 years after a mental disorder diagnosis, with comparable trends in the utilisation of psychological treatments. These findings on mental healthcare utilisation in the mid term to long term after CRC diagnosis also complement our 2024 qualitative study, where participants with CRC shared ongoing emotional and mental impacts extending into the mid-term to long-term after a CRC diagnosis.^14^ Altogether, our findings expand existing literature by demonstrating that disparities indeed persist in individuals with CRC, even after a formal mental disorder diagnosis. As a result, our current study suggests the importance of ensuring access to mental healthcare for individuals with CRC long after cancer diagnosis and treatment. Sustained mental healthcare throughout the spectrum of CRC—from diagnosis, treatment and into survivorship—can better address the enduring psychological challenges faced by individuals with CRC. By identifying these disparities, our study provides a foundation for future work to explore underlying reasons for lower treatment uptake and to develop targeted strategies that improve mental healthcare utilisation for this population.

Our study has several strengths, including the use of a large, population-based administrative health data that captures a diverse population and is directly generalisable to the population, particularly to the province of BC. Additionally, the use of administrative health data minimises recall bias, as our data are not based on participant responses or self-report. Lastly, the longitudinal nature of our data (from 1986 to 2017) allows for the assessment of trends and temporal relationships over time. There are also limitations to our study given the nature of our data source. While we used a robust dataset by linking Population Data BC with the BC Cancer Registry, it is important to acknowledge that our administrative health databases do not capture privately delivered mental services (eg, psychologists), or services directly available to patients with cancer through BC Cancer, the publicly funded agency that delivers comprehensive cancer care to all people in BC. Consequently, the receipt of minimally adequate psychological treatments is likely underestimated in our findings due to the limitation of our data in capturing privately funded mental health services. The administrative health data also lack variables on social determinants of health and the severity of anxiety and/or depression, both of which are important factors that may influence healthcare engagement and treatment decisions. Furthermore, the PharmaNet database used to evaluate pharmacotherapy only captures information on dispensed prescriptions and not unfilled prescriptions, medications used after dispensation and patient refusal of medications.

An implication of this study is informing directions for future research. Our study period is limited by the availability of the BC Cancer Registry data to 31 December 2018 based on our data access agreement. While treatment approaches for anxiety and depression have remained stable in recent years, longer follow-up periods are warranted in future studies examining mental healthcare utilisation in CRC and other cancers. Future research could explore specific pharmacotherapy doses, patients’ responses to mental health treatments, and the effectiveness of both publicly and privately delivered ongoing mental healthcare for anxiety and depression throughout the entirety of CRC care. Future research should also investigate factors that may influence mental healthcare utilisation among individuals with CRC. Understanding these factors may help identify potential barriers and disparities in access to care.

## Conclusions

Our population-based study provides valuable insights into the mental healthcare utilisation of individuals with CRC, specifically on the treatment of incident anxiety and depression. Our findings highlight that a considerable proportion of individuals with CRC do not meet the criteria for minimally adequate antidepressant pharmacotherapy and/or psychological treatment. Our findings also underscore the need to optimise long-term mental healthcare, as individuals with CRC continue to use mental health treatments even 10 years after their mental disorder diagnosis.

## supplementary material

10.1136/bmjonc-2024-000690 online supplemental file 1

10.1136/bmjonc-2024-000690 online supplemental file 2

10.1136/bmjonc-2024-000690 online supplemental file 3

10.1136/bmjonc-2024-000690 online supplemental file 4

10.1136/bmjonc-2024-000690 online supplemental file 5

10.1136/bmjonc-2024-000690 online supplemental file 6

## Data Availability

Data are available on reasonable request.
