# Supplementary material for: Mental healthcare utilisation among individuals with colorectal cancer: population-based cohort studies
Source: BMJ Oncol. 2025 Apr 1;4(1):e000690. doi: 10.1136/bmjonc-2024-000690 (PMC11962786; doi:10.1136/bmjonc-2024-000690)
Supplement: online supplemental file 1 [file bmjonc-4-1-s001.pdf]

Supplementary Table 1. Medication Codes for Antidepressants and Anxiolytic According to the Anatomical Therapeutic Chemical (ATC) Classification System.

ATC Classification	Chemical Class
**N05B**	**Anxiolytics**
N05BA	Benzodiazepine derivatives
N05BB	Diphenylmethane derivatives
N05BC	Carbamates
N05BD	Dibenzo-bicyclo-octadiene derivatives
N05BE	Azaspirodecanedione derivatives
N05BX	Other anxiolytics
**N06A**	**Antidepressants**
N06AA	Non-selective monoamine reuptake inhibitors
N06AB	Selective serotonin reuptake inhibitors
N06AF	Monoamine oxidase inhibitors, non-selective
N06AG	Monoamine oxidase A inhibitors
N06AX	Other antidepressants

Supplementary Table 2. Procedural codes for psychological treatments (psychiatry, counselling, psychotherapy, telehealth, and general practitioner).

Procedural code(s)	Definition
**Psychiatrist visits**	
00605	Emergency visit – psychiatry
00607	Office visit – psychiatry
00608	Hospital visit – psychiatry
00609	Home visit – psychiatry
00610	Individual consultation – psychiatry
00625	Individual repeat consultation – psychiatry
00699	Miscellaneous – psychiatry
**Counselling**	
12120, 00120, 15320, 16120, 17120, 18120	Individual counselling – in office
12220, 13220, 15220, 16220, 17220, 18220	Individual counselling – out of office
00121, 00122, 00513, 00515	Group counselling
00514	Prolonged visit for counselling
00276, 00277	Prolonged visit/or counselling complex case
**Psychotherapy**	

00630, 00631, 00632	Individual psychotherapy (office or hospital out-patient)
00650, 00651, 00652	Individual psychotherapy (hospital or institution in-patient or home)
00633, 00635, 00636, 00638, 00639	Family/Conjoint Psychotherapy
00663, 00664, 00665, 00666, 00667, 00668, 00669, 00670, 00671, 0000672, 00673, 00674, 00675, 00676, 00677, 00678, 00679, 00680, 00681	Group Psychotherapy
**Telehealth**	
13018	Telehealth GP in-office individual counselling
13021, 13022	Telehealth GP out-of-office group counselling
13038	Telehealth GP in-office individual counselling for prolonged visit
60607	Telehealth subsequent office visit – psychiatric treatment
60608	Telehealth hospital in-patient visit – psychiatric treatment
60610	Full telehealth consultation – psychiatry
60613	Telehealth geriatric consult – psychiatry

60625	Telehealth repeat or limited consult – psychiatry
60630, 60631, 60632	Individual telehealth psychiatric treatment
**GP mental health planning**	
G14044, G14045, G14046, G14047, G14048	GP Mental health management

Supplementary Table 3. Characteristics of study samples of individuals with early-age onset colorectal cancer (EAO-CRC) and no EAO-CRC matched on incident (1) anxiety, (2) depression, and (3) anxiety + depression.

	**Study Sample 1 Anxiety**		**Study Sample 2 Depression**		**Study Sample 3 Anxiety + Depression**	
**Characteristic**	**EAO-CRC (n=174)**	**No EAO-CRC (n=174)**	**EAO-CRC (n=477)**	**No EAO-CRC (n=477)**	**EAO-CRC (n=68)**	**No EAO-CRC (n=68)**
Years from CRC diagnosis to mental disorder diagnosis, mean	3.5	3.7	2.8	2.8	6.1	6.1
**Demographic factors**						
Age, mean (SD)	41.4 (6.6)	41.4 (6.6)	42.2 (6.3)	42.2 (6.3)	40.9 (7.0)	40.9 (7.0)
Sex, n (%)						
Male	66 (37.9)	66 (37.9)	240 (50.3)	240 (50.3)	32 (47.1)	32 (47.1)
Female	108 (62.07)	108 (62.07)	237 (50.0)	237 (50.0)	36 (52.9)	36 (52.9)
Neighbourhood income quintile, n (%)						
Quintile 1	33 (19.0)	28 (16.1)	102 (31.4)	110 (23.1)	15 (22.1)	15 (22.1)
Quintile 2	28 (16.1)	34 (19.5)	85 (17.8)	74 (15.5)	14 (20.6)	11 (16.2)
Quintile 3	32 (18.4)	47 (27.0)	101 (21.2)	103 (21.6)	10 (14.7)	9 (13.2)
Quintile 4	45 (25.9)	33 (19.0)	98 (20.6)	95 (19.9)	18 (26.5)	22 (32.4)
Quintile 5	36 (20.7)	32 (18.4)	91 (19.1)	95 (19.9)	11 (16.2)	11 (16.2)
Residence, n (%)						
Urban	150 (86.2)	156 (89.7)	426 (89.3)	412 (86.4)	59 (86.8)	59 (86.8)
Rural	24 (13.8)	18 (10.3)	51 (10.7)	65 (13.6)	9 (13.2)	9 (13.2)
**Healthcare utilization**						
Outpatient visits, mean (SD)	12.3 (10.0)	9.3 (9.2)	12.1 (12.7)	8.7 (12.0)	12.1 (7.4)	14.1 (18.3)
**Comorbidities**						
Charlson-Romano comorbidity index, mean (SD)[A]	1.2 (2.2)	0.1 (0.6)	1.5 (2.5)	0.1 (0.5)	1.3 (2.2)	0.2 (0.8)
**CRC characteristics**						

Site[B], n (%)						
Left colon	70 (40.2)	---	183 (38.4)	---	---	---
Right colon	14 (8.0)	---	47 (9.9)	---	---	---
Transverse colon	9 (5.2)	---	24 (5.0)	---	---	---
Unspecified	6 (3.4)	---	27 (5.7)	---	---	---
Rectum	75 (43.1)	---	196 (41.1)	---	---	---
Treatment, n (%)						
Surgery	125 (71.8)	---	299 (62.7)	---	48	---
Chemotherapy	113 (64.9)	---	283 (59.3)	---	43	---
Radiation	57 (32.8)	---	158 (33.1)	---	22	---
Stage[B,C], n (%)	**(n=49)**		**(n=73)**			
1	6 (12.2)	---	6 (8.2)	---	---	---
2	8 (16.3)	---	21 (28.8)	---	---	---
3	24 (49.0)	---	35 (47.9)	---	---	---
4	11 (22.4)	---	11 (15.1)	---	---	---

Descriptive statistics were determined for the year prior to EAO-CRC diagnosis date/matched date.
[A]All comorbidities except for cancer.
[B]Data for anxiety + depression is unavailable due to residual disclosure risk.
[C]Stage data available from 2010 onwards.
Abbreviations: EAO-CRC – early-age onset colorectal cancer; SD – standard deviation

Supplementary Table 4. Characteristics of study samples of individuals with average-age onset colorectal cancer (AAO-CRC) and no AAO-CRC matched on incident (1) anxiety, (2) depression, and (3) anxiety + depression.

	**Study Sample 1 Anxiety**		**Study Sample 2 Depression**		**Study Sample 3 Anxiety + Depression**	
**Characteristic**	**AAO-CRC (n=1,288)**	**No AAO-CRC (n=1,288)**	**AAO-CRC (n=4,163)**	**No AAO-CRC (n=4,163)**	**AAO-CRC (n=383)**	**No AAO-CRC (n=383)**
Years from CRC diagnosis to mental disorder diagnosis, median	3.9	3.9	3.8	3.8	7.7	7.7
**Demographic factors**						
Age, mean (SD)	67.7 (9.4)	67.7 (9.4)	69.1 (9.4)	69.1 (9.4)	68.1 (9.2)	68.1 (9.2)
Sex, n (%)						
Male	530 (41.2)	530 (41.2)	2,024 (48.6)	2,024 (48.6)	146 (38.1)	146 (38.1)
Female	758 (58.9)	758 (58.9)	2,139 (51.4)	2,139 (51.4)	237 (61.9)	237 (61.9)
Neighbourhood income quintile, n (%)						
Quintile 1	302 (23.5)	304 (23.6)	924 (22.2)	965 (23.2)	95 (24.8)	79 (20.6)
Quintile 2	249 (19.3)	226 (17.6)	802 (19.3)	814 (19.6)	71 (18.5)	87 (22.7)
Quintile 3	267 (20.7)	250 (19.4)	836 (20.1)	793 (19.1)	67 (17.5)	73 (19.1)
Quintile 4	232 (18.0)	256 (19.9)	786 (18.9)	834 (20.0)	68 (17.8)	68 (17.8)
Quintile 5	238 (18.5)	252 (19.6)	815 (19.6)	757 (18.2)	82 (21.4)	76 (19.8)
Residence, n (%)						
Urban	1,114 (86.5)	1,108 (86.0)	3,608 (86.7)	3,594 (86.3)	339 (88.5)	325 (84.9)
Rural	174 (13.5)	180 (14.0)	555 (13.3)	569 (13.7)	44 (11.5)	58 (15.1)
**Healthcare utilization**						
Outpatient visits, mean (SD)	16.3 (12.3)	13.7 (13.4)	14.8 (10.5)	11.9 (11.9)	16.3 (10.3)	14.7 (13.9)
**Comorbidities**						
Charlson-Romano comorbidity index, mean (SD)[A]	1.8 (2.5)	0.3 (0.9)	1.8 (2.4)	0.3 (0.9)	1.8 (2.5)	0.3 (0.7)
**CRC characteristics**						
Site[B], n (%)						

Left colon	542 (42.1)	---	1764 (41.4)	---	---	---
Right colon	210 (16.3)	---	572 (13.4)	---	---	---
Transverse colon	74 (5.7)	---	248 (5.8)	---	---	---
Unspecified	45 (3.5)	---	156 (3.7)	---	---	---
Rectum	417 (32.4)	---	1423 (33.4)	---	---	---
Treatment, n (%)						
Surgery	636 (49.4)	---	1943 (45.6)	---	181 (47.3)	---
Chemotherapy	515 (40.0)	---	1540 (36.1)	---	131 (34.2)	---
Radiation	274 (21.3)	---	928 (21.8)	---	72 (18.8)	---
Stage[B,C], n (%)	**(n=263)**		**(n=510)**			
1	61 (23.2)	---	106 (20.8)	---	---	---
2	71 (27.0)	---	144 (28.2)	---	---	---
3	96 (36.5)	---	182 (35.7)	---	---	---
4	35 (13.3)	---	78 (15.3)	---	---	---

Descriptive statistics were determined for the year prior to AAO-CRC diagnosis date/matched date.
[A]All comorbidities except for cancer.
[B]Data for anxiety + depression is unavailable due to residual disclosure risk.
[C]Stage data available from 2010 onwards.
Abbreviations: AAO-CRC – average-age onset colorectal cancer; SD – standard deviation

Supplementary Table 5. Utilization of mental health care in study samples of individuals with CRC and no CRC with (1) anxiety, (2) depression, and (3) anxiety + depression reported as numbers of encounters.

Outcome	Study Sample 1 Anxiety			Study Sample 2 Depression			Study Sample 3 Anxiety + Depression		
	CRC	No CRC	p-value	CRC	No CRC	p-value	CRC	No CRC	p-value
**Pharmacotherapy, mean (SD)**									
Number of anxiolytic prescriptions	2.8 (6.5)	2.9 (7.6)	**<0.05**	1.9 (8.1)	1.5 (4.6)	**<0.05**	3.7 (8.5)	3.7 (8.6)	0.66
Number of antidepressant prescriptions	4.4 (10.3)	5.8 (14.9)	**<0.05**	5.5 (14.0)	5.6 (12.8)	**<0.05**	6.9 (12.2)	9.2 (21.7)	0.19
**Psychological Treatment, mean (SD)**									
**By service type**									
Psychiatrist	0.4 (3.3)	0.5 (2.5)	**<0.05**	0.5 (2.4)	0.6 (3.0)	0.97	0.6 (3.2)	0.9 (3.5)	0.20
Publicly funded counselling	0.9 (1.5)	0.8 (1.2)	0.09	1.1 (1.4)	1.0 (1.4)	**<0.05**	0.9 (1.3)	0.8 (1.3)	0.67
Publicly funded psychotherapy	1.3 (6.1)	1.7 (7.9)	**<0.05**	1.1 (4.4)	1.1 (4.6)	0.19	2.0 (6.6)	2.2 (6.0)	0.21
Telehealth	0.00093 (0.03)	0.00083 (0.03)	0.94	0.0012 (0.1)	0.0011 (0.04)	0.75	0.012 (0.2)	0	0.13
Mental health planning	0.02 (0.2)	0.02 (0.2)	0.81	0.02 (0.2)	0.02 (0.3)	0.95	0.021 (0.2)	0.016 (0.1)	0.61
**Hospitalizations, mean (SD)**									
For anxiety	0.3 (0.5)	0.3 (0.5)	**<0.05**	0.02 (0.2)	0.02 (0.1)	0.92	0.3 (0.5)	0.3 (0.6)	0.07
For depression	0.1 (0.9)	0.2 (0.9)	0.34	0.2 (0.8)	0.1 (0.6)	0.0051	0.4 (2.1)	0.2 (0.6)	0.44
For anxiety + depression	0.1 (0.3)	0.1 (0.4)	0.13	0.01 (0.1)	0.02 (0.1)	0.19	0.1 (0.4)	0.2 (0.4)	0.91

Abbreviations: CRC – colorectal cancer; SD – standard deviation

Supplementary Table 6. Minimally adequate antidepressant pharmacotherapy and psychological treatment in study samples of individuals with CRC and no CRC with (1) anxiety, (2) depression, and (3) anxiety + depression.

Outcome	Study Sample 1 Anxiety			Study Sample 2 Depression			Study Sample 3 Anxiety + Depression		
	**CRC**	**No CRC**	**p-value**	**CRC**	**No CRC**	**p-value**	**CRC**	**No CRC**	**p-value**
**Minimally adequate antidepressant pharmacotherapy**									
Proportion of individuals, n (%)	390 (36.2)	507 (42.0)	**<0.05**	1,551 (46.3)	1,923 (50.9)	**<0.05**	190 (57.6)	234 (62.7)	0.16
Days' supply[A], mean (SD)	237.7 (126.6)	246.0 (128.5)	0.06	227.4 (128.0)	232.8 (124.8)	0.12	261.5 (116.6)	253.2 (117.4)	0.63
Proportion of days covered, mean (SD)	0.7 (0.3)	0.7 (0.01)	0.06	0.6 (0.4)	0.6 (0.3)	0.12	0.7 (0.3)	0.7 (0.3)	0.63
**Minimally adequate psychological treatment**									
Proportion of individuals, n (%)	171 (15.9)	212 (17.6)	0.28	672 (20.1)	701 (18.6)	0.11	78 (23.6)	97 (26.0)	0.53
All psychological treatment services, mean (SD)	2.7 (8.0)	3.0 (9.0)	0.71	2.7 (5.7)	2.7 (6.4)	**<0.05**	3.5 (8.0)	3.9 (7.8)	0.79
**Minimally adequate antidepressant pharmacotherapy OR psychological treatment**									
Proportion of individuals, n (%)	447 (41.5)	580 (48.1)	**<0.05**	1,883 (46.3)	2,233 (59.1)	**<0.05**	208 (63.0)	262 (70.2)	**<0.05**
**Minimally adequate antidepressant pharmacotherapy AND psychological treatment**									
Proportion of individuals, n (%)	114 (10.6)	139 (11.5)	0.47	340 (10.1)	391 (10.4)	0.78	60 (18.2)	69 (18.5)	0.91

[A]Days' supply refers to the mean days of supply of antidepressant prescriptions dispensed over 365 days for CRC and no CRC with at least one antidepressant dispensed.

Abbreviations: CRC – colorectal cancer; SD – standard deviation

Supplementary Table 7. Utilization of mental health care in study samples of individuals with EAO-CRC and no EAO-CRC with (1) anxiety, (2) depression, and (3) anxiety + depression reported as proportion among individuals.

Outcome	Study Sample 1 Anxiety			Study Sample 2 Depression			Study Sample 3 Anxiety + Depression		
	**EAO-CRC**	**No EAO-CRC**	**p-value**	**EAO-CRC**	**No EAO-CRC**	**p-value**	**EAO-CRC**	**No EAO-CRC**	**p-value**
**Pharmacotherapy, n (%)**									
**Anxiolytics**									
All anxiolytics	59 (43.7)	59 (38.1)	**<0.05**	100 (26.3)	105 (24.5)	**<0.05**	25 (49.0)	29 (46.8)	0.85
**By class**									
Benzodiazepines	57 (42.2)	49 (31.6)	0.07	114 (30.0)	102 (23.8)	**<0.05**	23 (45.1)	26 (41.9)	0.85
Hydroxyzine	<5[C]	<5[C]	0.38	7 (1.8)	<5[C]	0.36	<5[C]	<5[C]	0.38
Buspirone	<5[C]	<5[C]	0.60	0	5 (1.2)	0.06	<5[C]	<5[C]	1.00
**Antidepressants**									
All antidepressants	71 (52.6)	80 (51.6)	0.91	207 (54.5)	276 (64.3)	**<0.05**	33 (64.7)	49 (79.0)	0.10
**By class**									
Selective serotonin reuptake inhibitors (SSRI)	49 (36.3)	61 (39.4)	0.63	133 (35.0)	207 (48.3)	**<0.05**	23 (45.1)	30 (48.4)	0.85
Non-selective monoamine reuptake inhibitors[A]	14 (10.4)	12 (7.7)	0.54	39 (10.3)	35 (8.2)	0.33	6 (11.8)	7 (11.3)	1.00
Other[B]	30 (22.2)	29 (18.7)	0.47	75 (19.7)	100 (23.3)	0.23	17 (33.3)	30 (48.4)	0.13
**Psychological Treatment, n (%)**									
**By service type**									
Psychiatrist	12 (8.9)	26 (16.8)	0.06	67 (17.6)	79 (18.4)	0.78	10 (19.6)	16 (25.8)	0.50
Publicly funded counselling	68 (50.4)	73 (47.1)	0.64	261 (68.7)	294 (68.5)	1.00	28 (54.9)	27 (43.5)	0.26
Publicly funded psychotherapy	18 (13.3)	34 (21.9)	0.07	63 (16.6)	61 (14.2)	0.38	10 (19.6)	20 (32.3)	0.14
Telehealth	<5[C]	<5[C]	1.00	0	0	1.00	<5[C]	0	0.45
Mental health planning	<5[C]	<5[C]	0.34	5 (1.3)	7 (1.6)	0.78	<5[C]	0	0.20
**Hospitalizations, n (%)**									
For anxiety	25 (18.5)	20 (12.9)	0.20	<5[C]	<5[C]	0.35	11 (21.6)	13 (21.0)	1.00
For depression	9 (6.7)	16 (10.3)	0.30	31 (8.2)	19 (4.4)	**<0.05**	6 (11.8)	11 (17.7)	0.44
For anxiety + depression	8 (5.9)	15 (9.7)	0.28	<5[C]	<5[C]	0.35	5 (9.8)	9 (14.5)	0.57

[A]Include tricyclic antidepressants.
[B]Other antidepressants included selective serotonin-norepinephrine reuptake inhibitors, trazodone, and mirtazapine.
[C]Cell sizes <5 not reported according to agreements of the data access request.
Abbreviations: EAO-CRC – early-age onset colorectal cancer; SD – standard deviation

Supplementary Table 8. Utilization of mental health care in study samples of individuals with AAO-CRC and no AAO-CRC with (1) anxiety, (2) depression, and (3) anxiety + depression reported as proportion among individuals.

Outcome	Study Sample 1 Anxiety			Study Sample 2 Depression			Study Sample 3 Anxiety + Depression		
	**AAO-CRC**	**No AAO-CRC**	**p-value**	**AAO-CRC**	**No AAO-CRC**	**p-value**	**AAO-CRC**	**No AAO-CRC**	**p-value**
**Pharmacotherapy, n (%)**									
**Anxiolytics**									
All anxiolytics	434 (46.0)	435 (41.3)	**<0.05**	989 (33.3)	1,054 (31.5)	0.15	136 (48.7)	148 (47.6)	0.80
**By class**									
Benzodiazepines	424 (45.0)	430 (40.9)	0.07	941 (31.7)	1,000 (29.9)	0.12	135 (48.4)	143 (46.0)	0.56
Hydroxyzine	16 (1.7)	20 (1.9)	0.87	71 (2.4)	75 (2.2)	0.74	6 (2.2)	7 (2.3)	1.00
Buspirone	<5[C]	5 (0.5)	1.00	5 (0.2)	12 (0.4)	0.22	0	<5[C]	0.13
**Antidepressants**									
All antidepressants	415 (44.0)	545 (51.8)	**<0.05**	1,767 (59.5)	2,084 (62.2)	**<0.05**	186 (66.7)	219 (70.4)	0.33
**By class**									
Selective serotonin reuptake inhibitors (SSRI)	285 (30.2)	336 (31.9)	0.41	1,242 (41.8)	1,507 (45.0)	**<0.05**	138 (49.5)	127 (40.8)	**<0.05**
Non-selective monoamine reuptake inhibitors[A]	72 (7.6)	90 (8.6)	0.46	239 (8.0)	292 (8.7)	0.34	27 (9.7)	32 (10.3)	0.89
Other[B]	168 (17.8)	252 (24.0)	**<0.05**	668 (22.5)	765 (22.8)	0.74	71 (25.4)	123 (39.5)	**<0.05**
**Psychological Treatment, n (%)**									
**By service type**									
Psychiatrist	81 (8.6)	122 (11.6)	**<0.05**	388 (13.1)	429 (12.8)	0.79	38 (13.6)	50 (16.1)	0.42
Publicly funded counselling	406 (43.1)	417 (39.6)	0.13	1,435 (48.3)	1,449 (43.3)	**<0.05**	128 (45.9)	124 (39.9)	0.16
Publicly funded psychotherapy	124 (13.1)	175 (16.6)	**<0.05**	538 (18.1)	568 (17.0)	0.23	67 (24.0)	84 (27.0)	0.45
Telehealth	0	0	1.00	<5[C]	<5[C]	1.00	<5[C]	0	0.47
Mental health planning	6 (0.6)	8 (0.8)	0.79	30 (1.0)	33 (1.0)	1.00	<5[C]	5 (1.6)	1.00
**Hospitalizations, n (%)**									
For anxiety	264 (28.0)	243 (23.1)	**<0.05**	52 (1.8)	60 (1.8)	0.92	86 (30.8)	73 (23.5)	0.05
For depression	84 (8.9)	102 (9.7)	0.59	443 (14.9)	428 (12.8)	**<0.05**	63 (22.6)	58 (18.6)	0.26
For anxiety + depression	66 (7.0)	88 (8.4)	0.28	34 (1.1)	54 (1.6)	0.13	40 (14.3)	40 (12.9)	0.63

[A]Include tricyclic antidepressants.
[B]Other antidepressants included selective serotonin-norepinephrine reuptake inhibitors, trazodone, and mirtazapine.
[C]Cell sizes <5 not reported according to agreements of the data access request.
Abbreviations: AAO-CRC – average-age onset colorectal cancer; SD – standard deviation

Supplementary Table 9. Utilization of mental health care in study samples of individuals with EAO-CRC and no EAO-CRC with (1) anxiety, (2) depression, and (3) anxiety + depression reported as numbers of encounters.

**Outcome**	**Study Sample 1 Anxiety**			**Study Sample 2 Depression**			**Study Sample 3 Anxiety + Depression**		
	**EAO-CRC**	**No EAO-CRC**	**p-value**	**EAO-CRC**	**No EAO-CRC**	**p-value**	**EAO-CRC**	**No EAO-CRC**	**p-value**
**Pharmacotherapy, mean (SD)**									
Number of anxiolytic prescriptions	2.8 (9.1)	1.7 (4.8)	**<0.05**	1.6 (5.0)	0.7 (2.1)	**<0.05**	4.3 (13.7)	2.1 (4.1)	0.90
Number of antidepressant prescriptions	4.5 (13.1)	3.5 (5.1)	0.96	3.0 (5.5)	4.6 (18.2)	**<0.05**	6.7 (18.9)	10.8 (44.0)	0.18
**Psychological Treatment, mean (SD)**									
**By service type**									
Psychiatrist	0.1 (0.7)	0.3 (1.2)	**<0.05**	0.3 (0.7)	0.4 (1.4)	0.76	0.3 (1.0)	0.5 (1.6)	0.37
Publicly funded counselling	1.1 (1.4)	1.0 (1.4)	0.55	1.5 (1.5)	1.5 (1.5)	0.83	1.2 (1.5)	1.1 (1.6)	0.44
Publicly funded psychotherapy	1.3 (5.4)	2.0 (6.1)	0.06	1.4 (6.0)	1.2 (4.6)	0.40	1.9 (9.7)	2.4 (6.3)	0.10
Telehealth	0.0074 (0.1)	0.0065 (0.1)	0.93	0	0	1.00	0.02 (0.1)	0	0.28
Mental health planning	0.04 (0.3)	0.02 (0.2)	0.26	0.02 (0.1)	0.03 (0.3)	0.71	0.1 (0.3)	0	0.12
**Hospitalizations, mean (SD)**									
For anxiety	0.2 (0.4)	0.2 (0.4)	0.20	0.0079 (0.1)	0.0023 (0.048)	0.26	0.2 (0.5)	0.2 (0.5)	0.97
For depression	0.1 (0.3)	0.1 (0.3)	0.27	0.1 (0.3)	0.1 (0.3)	**<0.05**	0.1 (0.4)	0.2 (0.4)	0.41
For anxiety + depression	0.1 (0.2)	0.1 (0.3)	0.24	0.0079 (0.1)	0.0023 (0.05)	0.26	0.1 (0.3)	0.1 (0.4)	0.45

Abbreviations: EAO-CRC – early-age onset colorectal cancer; SD – standard deviation

Supplementary Table 10. Utilization of mental health care in study samples of individuals with AAO-CRC and no AAO-CRC with (1) anxiety, (2) depression, and (3) anxiety + depression reported as numbers of encounters.

Outcome	Anxiety			Depression			Anxiety + Depression		
	**AAO-CRC**	**No AAO-CRC**	**p-value**	**AAO-CRC**	**No AAO-CRC**	**p-value**	**AAO-CRC**	**AAO-CRC**	**p-value**
**Pharmacotherapy, mean (SD)**									
Number of anxiolytic prescriptions	2.8 (6.0)	3.0 (7.9)	0.06	1.9 (8.4)	1.6 (4.9)	0.06	3.6 (7.2)	4.1 (9.2)	0.71
Number of antidepressant prescriptions	4.4 (9.8)	6.1 (15.8)	**<0.05**	5.8 (14.8)	5.7 (11.9)	**<0.05**	6.9 (10.6)	8.9 (13.6)	0.37
**Psychological Treatment, mean (SD)**									
**By service type**									
Psychiatrist	0.5 (3.5)	0.5 (2.6)	**<0.05**	0.5 (2.5)	0.6 (3.1)	0.92	0.6 (3.5)	1.0 (3.7)	0.33
Publicly funded counselling	0.9 (1.5)	0.8 (1.2)	0.11	1.0 (1.4)	0.9 (1.3)	**<0.05**	0.9 (1.2)	0.7 (1.2)	0.09
Publicly funded psychotherapy	1.3 (6.2)	1.7 (8.1)	0.04	1.1 (4.2)	1.1 (4.6)	0.26	2.0 (5.9)	2.1 (6.0)	0.49
Telehealth	0	0	1.00	0.0013 (0.1)	0.0012 (0.04)	0.75	0.01075 (0.2)	0	0.29
Mental health planning	0.02 (0.2)	0.02 (0.2)	0.74	0.02 (0.2)	0.02 (0.3)	0.93	0.0143 (0.1)	0.01929 (0.2)	0.86
**Hospitalizations, mean (SD)**									
For anxiety	0.2 (0.5)	0.3 (0.5)	**<0.05**	0.02 (0.2)	0.02 (0.2)	0.90	0.3 (0.6)	0.3 (0.6)	0.06
For depression	0.2 (0.9)	0.2 (0.9)	0.54	0.2 (0.8)	0.2 (0.6)	**<0.05**	0.4 (2.3)	0.3 (0.6)	0.27
For anxiety + depression	0.1 (0.3)	0.1 (0.4)	0.24	0.01 (0.1)	0.02 (0.1)	0.11	0.2 (0.4)	0.2 (0.4)	0.67

Abbreviations: AAO-CRC – average-age onset colorectal cancer; SD – standard deviation

Supplementary Table 11. Minimally adequate antidepressant pharmacotherapy and psychological treatment in study samples of individuals with EAO-CRC and no EAO-CRC with (1) anxiety, (2) depression, and (3) anxiety + depression.

Outcome	Study Sample 1 Anxiety			Study Sample 2 Depression			Study Sample 3 Anxiety + Depression		
	**EAO-CRC**	**No EAO-CRC**	**p-value**	**EAO-CRC**	**No EAO-CRC**	**p-value**	**EAO-CRC**	**No EAO-CRC**	**p-value**
**Minimally adequate antidepressant pharmacotherapy**									
Proportion of individuals, n (%)	57 (42.2)	61 (39.4)	0.62	150 (39.5)	216 (50.3)	**<0.05**	27 (52.9)	42 (67.7)	0.11
Days' supply[A], mean (SD)	228.5 (128.1)	231.8 (131.7)	0.78	203.7 (131.9)	205.6 (123.2)	0.80	233.8 (127.3)	225.7 (123.9)	0.84
Proportion of days covered, mean (SD)	0.6 (0.4)	0.6 (0.4)	0.78	0.6 (0.4)	0.6 (0.3)	0.80	0.6 (0.3)	0.6 (0.3)	0.84
**Minimally adequate psychological treatment**									
Proportion of individuals, n (%)	25 (18.5)	36 (23.2)	0.33	95 (25.0)	108 (25.2)	0.89	12 (23.5)	20 (32.3)	0.31
All psychological treatment services, mean (SD)	2.6 (5.6)	3.3 (6.6)	0.75	3.1 (6.3)	3.1 (5.8)	0.90	3.5 (9.8)	4.0 (7.1)	0.77
**Minimally adequate antidepressant pharmacotherapy OR psychological treatment**									
Proportion of individuals, n (%)	65 (48.1)	77 (49.7)	0.80	204 (53.7)	265 (61.8)	**<0.05**	30 (58.8)	48 (77.4)	**<0.05**
**Minimally adequate antidepressant pharmacotherapy AND psychological treatment**									
Proportion of individuals, n (%)	17 (12.6)	20 (12.9)	0.94	40 (10.5)	59 (13.8)	0.16	9 (17.6)	14 (22.6)	0.52

[A]Days' supply refers to the mean days of supply of antidepressant prescriptions dispensed over 365 days for EAO-CRC and no EAO-CRC with at least one antidepressant dispensed.

Abbreviations: EAO-CRC – early-age onset colorectal cancer; SD – standard deviation

Supplementary Table 12. Minimally adequate antidepressant pharmacotherapy and psychological treatment in study samples of individuals with AAO-CRC and no AAO-CRC with (1) anxiety, (2) depression, and (3) anxiety + depression.

Outcome	Study Sample 1 Anxiety			Study Sample 2 Depression			Study Sample 3 Anxiety + Depression		
	AAO-CRC	No AAO-CRC	p-value	AAO-CRC	No AAO-CRC	p-value	AAO-CRC	No AAO-CRC	p-value
**Minimally adequate antidepressant pharmacotherapy**									
Proportion of individuals, n (%)	333 (35.3)	446 (42.4)	**<0.05**	1,401 (47.2)	1,707 (51.0)	**<0.05**	163 (58.4)	192 (61.7)	0.41
Days' supply[A], mean (SD)	239.3 (126.4)	248.1 (128.1)	0.06	230.2 (127.3)	236.4 (124.6)	0.09	266.4 (114.3)	259.1 (115.4)	0.73
Proportion of days covered, mean (SD)	0.7 (0.3)	0.7 (0.4)	0.06	0.6 (0.3)	0.6 (0.3)	0.09	0.7 (0.3)	0.7 (0.3)	0.73
**Minimally adequate psychological treatment**									
Proportion of individuals, n (%)	146 (15.5)	176 (16.7)	0.45	578 (19.5)	593 (17.7)	0.07	66 (23.7)	77 (24.8)	0.75
All psychological treatment services, mean (SD)	2.7 (8.3)	3.0 (9.3)	0.7692	2.6 (5.7)	2.7 (6.5)	**<0.05**	3.5 (7.7)	3.8 (7.9)	0.7136
**Minimally adequate antidepressant pharmacotherapy OR psychological treatment**									
Proportion of individuals, n (%)	382 (40.5)	503 (47.8)	**<0.05**	1,679 (56.5)	1,968 (58.8)	0.07	178 (63.8)	214 (68.8)	0.20
**Minimally adequate antidepressant pharmacotherapy AND psychological treatment**									
Proportion of individuals, n (%)	97 (10.3)	119 (11.3)	0.46	300 (10.1)	332 (9.9)	0.81	51 (18.3)	55 (17.7)	0.85

[A]Days' supply refers to the mean days of supply antidepressant prescriptions dispensed over 365 days for AAO-CRC and no AAO-CRC with at least one antidepressant dispensed.

Abbreviations: AAO-CRC – average-age onset colorectal cancer; SD – standard deviation
