# Supplementary material for: Mental healthcare utilisation among individuals with colorectal cancer: population-based cohort studies
Source: BMJ Oncol. 2025 Apr 1;4(1):e000690. doi: 10.1136/bmjonc-2024-000690 (PMC11962786; doi:10.1136/bmjonc-2024-000690)
Supplement: online supplemental file 2 [file bmjonc-4-1-s002.pdf]

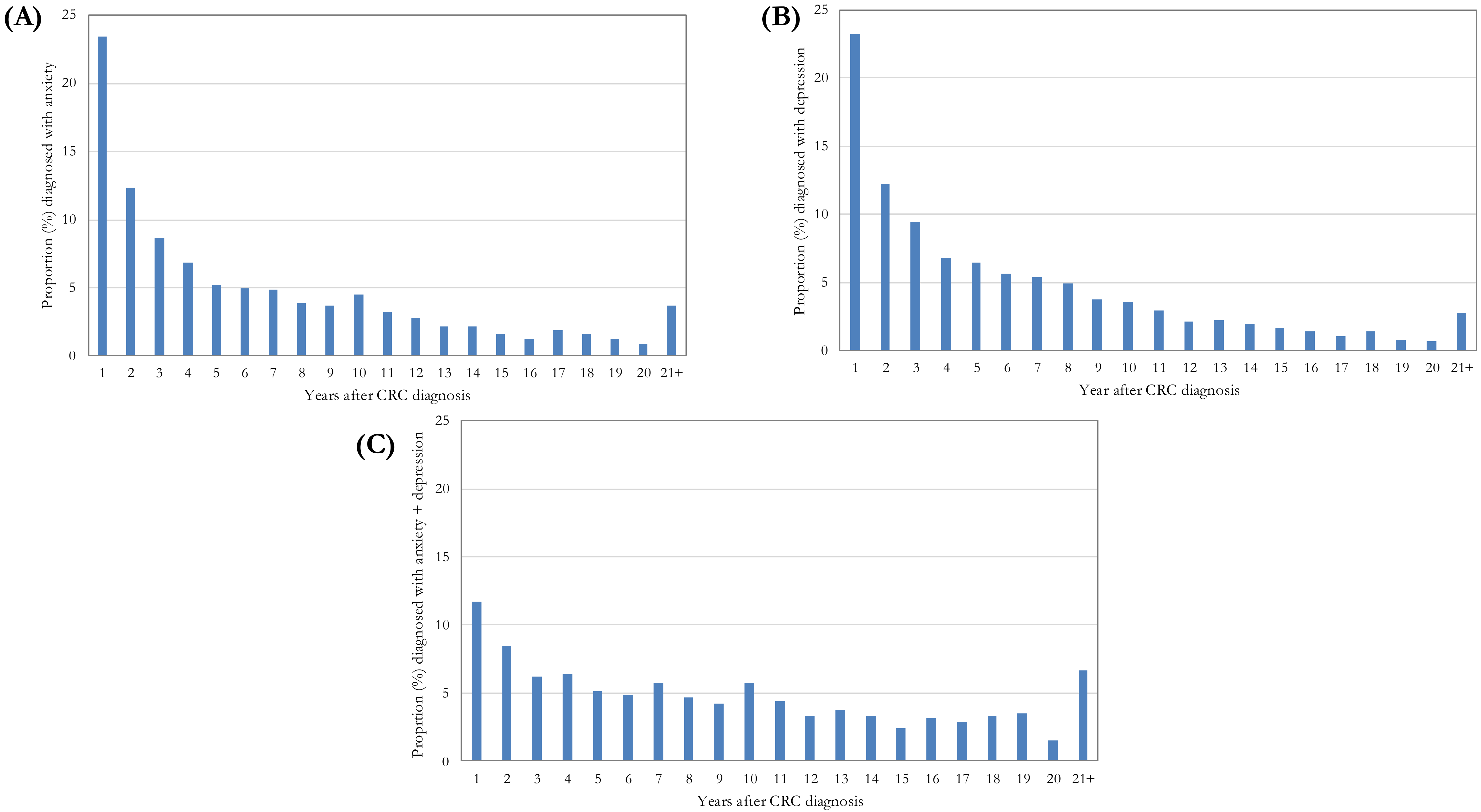

Supplementary Figure 1. Bar charts illustrating the time of diagnosis of (A) anxiety; (B) depression; (C) anxiety and depression among individuals with colorectal cancer (CRC).
