# Supplementary material for: Mental healthcare utilisation among individuals with colorectal cancer: population-based cohort studies
Source: BMJ Oncol. 2025 Apr 1;4(1):e000690. doi: 10.1136/bmjonc-2024-000690 (PMC11962786; doi:10.1136/bmjonc-2024-000690)
Supplement: online supplemental file 4 [file bmjonc-4-1-s004.pdf]

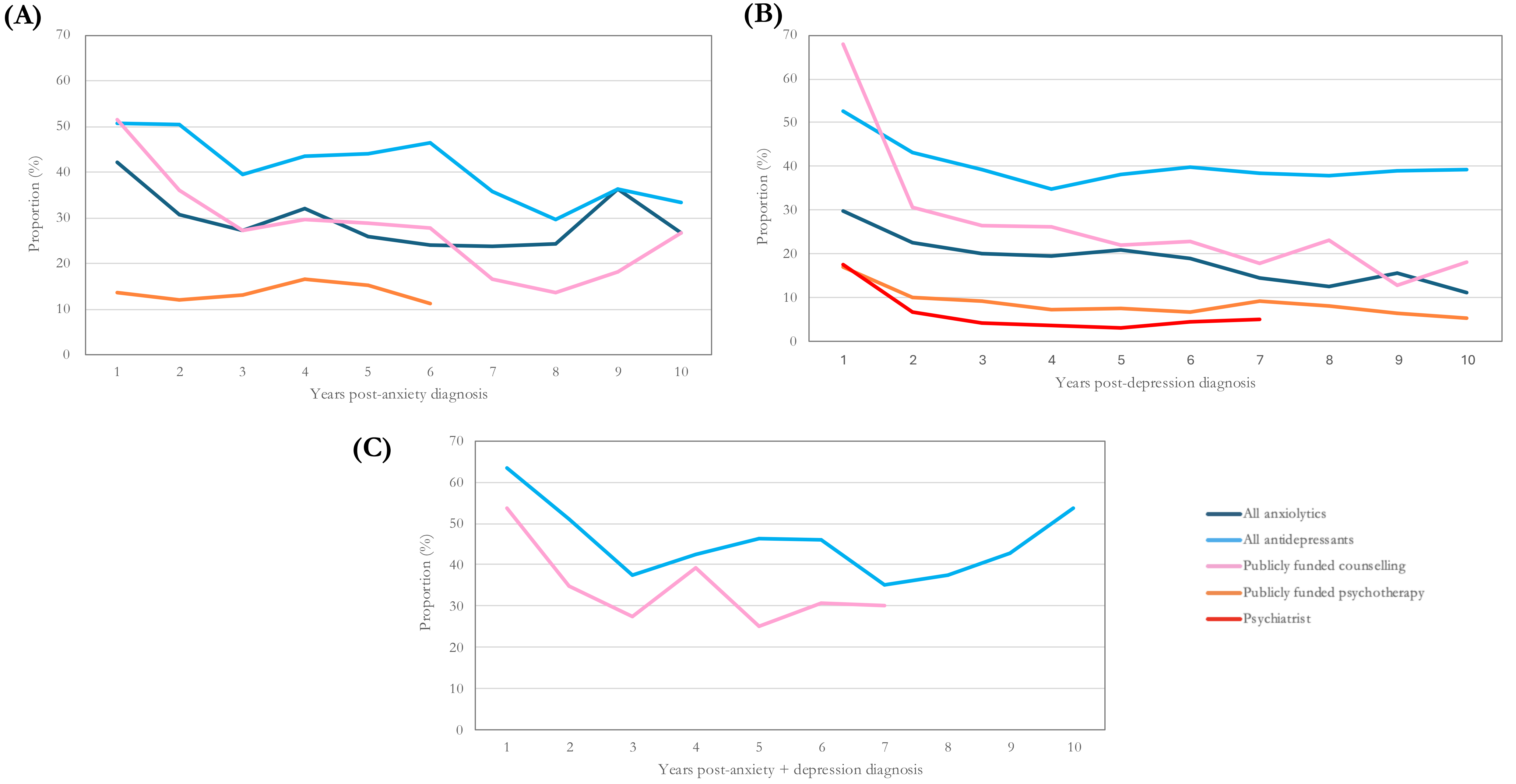

Supplementary Figure 3. Line graphs illustrating the proportion of mental health care utilization in years 1 to 10 after a diagnosis of (A) anxiety; (B); depression; (C) anxiety and depression among individuals with early-age onset colorectal cancer (EAO-CRC).
