# Supplementary material for: Mental healthcare utilisation among individuals with colorectal cancer: population-based cohort studies
Source: BMJ Oncol. 2025 Apr 1;4(1):e000690. doi: 10.1136/bmjonc-2024-000690 (PMC11962786; doi:10.1136/bmjonc-2024-000690)
Supplement: online supplemental file 6 [file bmjonc-4-1-s006.pdf]

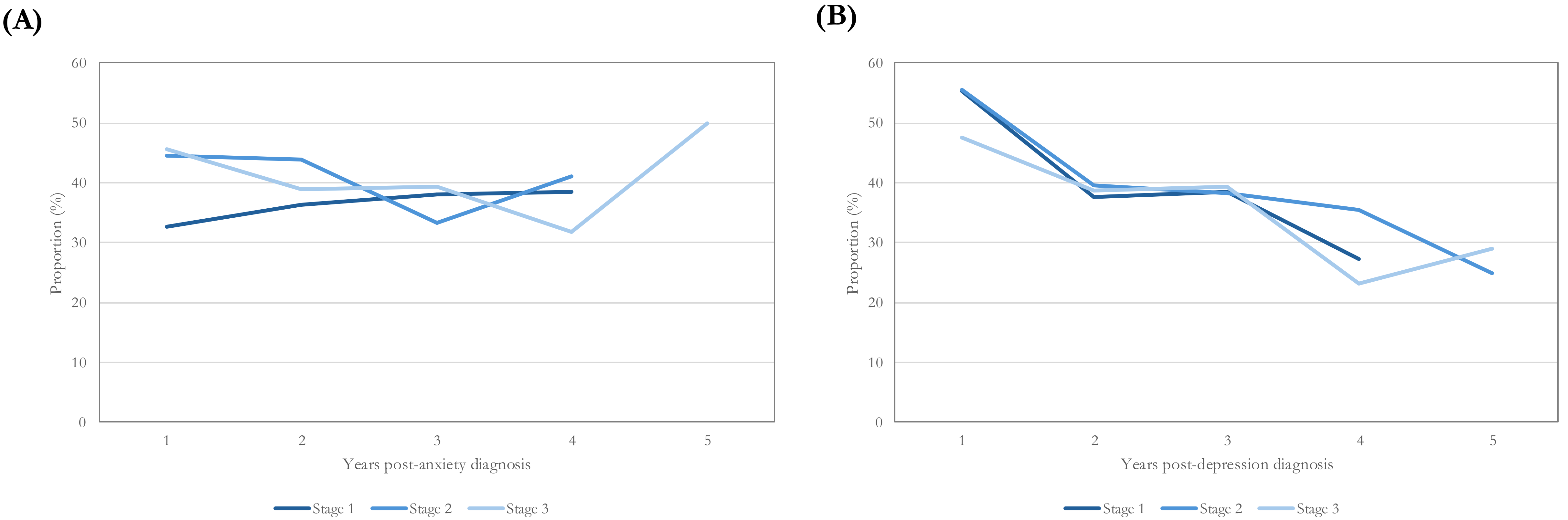

Supplementary Figure 5. Line graphs showing subgroup analysis by non-metastatic CRC (stages 1 to 3) at CRC diagnosis, illustrating the proportion of antidepressant utilization in years 1 to 5 after a diagnosis of (A) anxiety; (B); depression among individuals with colorectal cancer (CRC). Data for years 6 to 10 was unavailable due to residual disclosure risk.
